# From ER and mitochondria to contact sites: integrated regulation of ferroptosis in cardiovascular diseases

**DOI:** 10.1080/13510002.2026.2732768

**Published:** 2026-11-01

**Authors:** Sara Leo, Fabio Mollica, Gaia Pedriali, Giampaolo Morciano, Elena Nicoletta Colarusso, Elena Tremoli, Paolo Pinton

**Affiliations:** a IRCCS Maria Cecilia Hospital, GVM Care & Research, Cotignola, Italy; b Department of Biosciences, Biotechnologies and Environment, University of Bari “A. Moro”, Bari, Italy; c Department of Medical Sciences, Section of Experimental Medicine and Technopole of Ferrara, Laboratory for Advanced Therapies (LTTA), University of Ferrara, Ferrara, Italy

**Keywords:** Iron homeostasis, ferroptosis, lipid peroxidation, mitochondria, endoplasmic reticulum, mitochondria-associated membranes, organelle crosstalk, cardiovascular diseases

## Abstract

Iron is a central regulator of cellular redox homeostasis, supporting essential physiological processes. Given its critical biological importance, iron homeostasis is tightly regulated at both the systemic and cellular levels. Ferroptosis is a metabolically regulated, iron-dependent form of cell death characterized by the accumulation of phospholipid hydroperoxides driven by iron-catalyzed redox reactions, ultimately leading to oxidative membrane damage. Accumulating evidence indicates that ferroptosis emerges from the coordinated activity of multiple subcellular organelles rather than from isolated pathways. In this context, the endoplasmic reticulum (ER) and mitochondria have recently been established as key regulators of ferroptotic signaling. Notably, mitochondria-associated membranes (MAMs), by spatially coordinating redox signaling and lipid peroxidation, calcium fluxes, mitochondrial metabolism and lipid remodeling, are increasingly recognized as central hubs within the ferroptotic cascade, acting as modulators of cellular susceptibility to ferroptosis and as key sites of its execution. Owing to its strong dependence on mitochondrial oxidative phosphorylation, the heart is particularly sensitive to perturbations in iron homeostasis. Notably, growing evidence supports a pathogenic role for ferroptosis in cardiovascular diseases (CVDs), linking iron-dependent oxidative stress to cardiomyocyte dysfunction and tissue injury. Accordingly, ferroptosis has emerged as a potential therapeutic target in cardiovascular pathology. This review examines how ER and mitochondrial dysfunction contribute to ferroptotic signaling in CVDs and discusses the hypothesis that MAMs may function as central regulatory interfaces coordinating these processes. Elucidating these mechanisms is essential for the development of novel therapeutic strategies aimed at modulating ferroptosis and limiting CVDs progression.

## 1. Introduction

Iron is indispensable for a wide range of biological processes, serving a crucial role in fundamental cellular functions such as oxygen transport, DNA synthesis, and energy production via the Krebs cycle and electron transport chain [1]. Owing to its predominant dependence on aerobic mitochondrial ATP production, the heart is highly sensitive to disruptions in iron homeostasis. While iron deficiency is associated with various pathological conditions, including heart failure and its poorer prognosis [2], excessive iron accumulation can promote the generation of reactive oxygen species (ROS), leading to cellular dysfunction and cardiomyopathies [3].

Ferroptosis is a metabolically regulated, iron-dependent form of cell death that results from the dysregulation of intracellular and systemic iron homeostasis [4]. Although its susceptibility is influenced by specific genes, ferroptosis is not considered a genetically programmed cell death pathway, as it lacks a dedicated genome-encoded execution machinery comparable to that of apoptosis. Instead, it is biochemically, morphologically, and molecularly distinct from other forms of regulated cell death [4,5], and it is mechanistically defined by the iron-catalyzed accumulation of phospholipid hydroperoxides, leading to oxidative membrane damage. Within eukaryotic cells, several subcellular organelles cooperate to modulate ferroptosis and a growing body of evidence has highlighted mitochondria and the endoplasmic reticulum (ER) as key organelles orchestrating the ferroptotic pathway [6].

Far from being pure structural scaffolds, mitochondria-associated membranes (MAMs) integrate Ca^2+^ signaling, lipid metabolism, and mitochondrial homeostasis. Notably, recent findings have identified MAMs as early loci of lipid peroxidation and critical regulators of mitochondrial lipid radicals trafficking [7,8]. While these adaptations are consistent with the potential contribution of MAMs to ferroptotic regulation, it remains to be established whether they directly influence ferroptotic execution or instead reflect broader cellular stress responses.

The relevance of ferroptosis to cardiovascular diseases (CVDs) is underscored by the particular vulnerability of the heart to iron overload [9]. Among all organs, the heart’s exceptionally high energy demand, largely met through aerobic metabolism, predisposes it to elevated ROS production, thereby linking ferroptotic mechanisms more closely to cardiac pathology.

This review provides a comprehensive overview of recent advances in understanding the role of ferroptosis in CVDs, with a focus on mitochondrial and ER dysfunction. At the same time, we propose a conceptual framework in which MAMs act both as sensors of ferroptotic stimuli originating from the two juxtaposed organelles and as modulators of this regulated cell death pathway. A comprehensive understanding of the related molecular pathways is crucial for the identification of novel therapeutic and preventive strategies aimed at modulating ferroptotic signaling in CVDs, thereby mitigating cellular injury and improving clinical outcomes. In this context, a deeper mechanistic understanding of MAMs under ferroptotic conditions may open new avenues for the development of more effective therapeutic interventions.

## 2. Iron metabolism

### 2.1. Iron homeostasis

Iron is essential for a wide array of biological processes. In humans, most of the iron is incorporated into hemoglobin (72%), with additional amounts found in myoglobin within muscle cells, in storage proteins such as ferritin (FTH) and hemosiderin, and as cofactors in non-heme enzymes [1,10,11]. Dietary iron constitutes the main source of systemic iron. Iron is primarily absorbed by epithelial cells in the duodenum and upper jejunum, either directly in its soluble ferrous form (Fe^2+^) or, more frequently, when it is present as insoluble ferric iron (Fe^3+^) after being reduced to Fe^2+^ by the ferrireductase enzyme DcytB (Cybrd1) [12]. Conversely, heme iron (iron protoporphyrin IX) absorption into intestinal epithelial cells is thought to occur via endocytic and transporter-mediated pathways, followed by intracellular degradation by heme oxygenase 1 (HMOX1) [1,13,14]. In the bloodstream, iron primarily exists as Fe^3+^ associated with transferrin (Tf), which carries it to various organs. Consequently, cellular iron uptake in cardiomyocytes is primarily mediated by transferrin receptor 1 (TfR1) through receptor-mediated endocytosis. In mice, cardiac-specific TfR1 deficiency causes severe iron deprivation, resulting in mitochondrial dysfunction, hypertrophy, and ultimately, death [15]. Interestingly, transferrin receptor 2 (TfR2), a lower-affinity homolog of TfR1, is also expressed in the heart, and the genetic deletion of its *β* isoform has been shown to confer cardioprotection during myocardial ischemia/reperfusion (I/R) injury [16]. Once in the cell, the Tf-Fe^3+^ -TfR complex enters the endosome, where the acidic environment triggers the release of iron from Tf and its reduction to its highly reactive Fe^2+^ by members of the six-transmembrane epithelial antigen of the Prostate (STEAP) family of reductases [17,18]. The resulting Fe^2+^ is then transported into the cytosol by divalent metal transporter 1 (DMT1) and binds to FTH, a molecule composed of light (FTL) and heavy chains (FTH1), with the latter able to oxidize and store Fe^2+^, or enters the cytosolic labile iron pool (LIP), a reservoir of loosely chelated Fe^2+^, biologically available for cellular processes [10,19]. Interestingly, it was demonstrated that the selective silencing of FTH1 expression in cardiomyocytes is responsible for heart damage [20]. Meanwhile, Tf and TfR1 are largely recycled back to the cell surface. Ferroportin (FPN) is the only known iron exporter in mammals and mediates cellular iron efflux [10].

Despite extensive characterization of systemic and cellular iron handling, the mechanisms governing subcellular iron distribution, particularly in the mitochondria, remain incompletely understood. Mitochondria play a central role in cellular iron metabolism, contributing to the biosynthesis, utilization, and storage of iron–sulfur (Fe─S) clusters (ISC), which are essential for electron transport chain (ETC) activity and metabolic enzyme function. Upon entry into mitochondria, Fe^2+^ can be buffered by mitochondrial ferritin (mtFTH1), thereby limiting its redox activity, or remain in a metabolically available form within the matrix, constituting the mitochondrial labile iron pool (mtLIP) [21]. The molecular mechanisms governing iron translocation across the mitochondrial outer membrane (OMM) have not been fully elucidated, and iron permeation through the voltage-dependent anion channel (VDAC) has been proposed as a potential pathway [22]. Although some evidence implicates the mitochondrial calcium uniporter (MCU) in iron import into the mitochondrial matrix [23–25], mitochondrial iron uptake through the inner mitochondrial membrane (IMM) is most robustly and consistently attributed to mitoferrin proteins with SLC25A37/mitoferrin-1 primarily expressed in erythroid cells and SLC25A28/mitoferrin-2 ubiquitously expressed across non-erythroid tissues [26]. Modulation of mitoferrin expression has been shown to alter mitochondrial iron homeostasis across various pathological conditions [27–29]. Beyond these canonical mitochondrial iron transporters, emerging evidence suggests that iron distribution between the cytosol and mitochondria in cardiomyocytes is additionally regulated by the ER/Golgi-resident zinc–iron transporter ZIP13 [30]. Cardiac-specific deletion of ZIP13 disrupts intracellular iron homeostasis, resulting in reduced mitochondrial iron content accompanied by cytosolic iron accumulation in cardiomyocytes. The mechanisms governing mitochondrial iron export remain poorly understood. Notably, genetic deletion of the mitochondrial ATP-binding cassette transporter ABCB8 in cardiomyocytes results in mitochondrial iron accumulation and the development of cardiomyopathy, implicating ABCB8 as an important regulator of mitochondrial iron homeostasis, potentially through the modulation of iron efflux pathways [31].

### 2.2. Iron homeostasis regulation

Tight regulation of iron homeostasis, at both the systemic and cellular levels, is critical for supporting all iron-dependent physiological functions while minimizing the potential cytotoxicity associated with iron overload [32]. Systemically, the key iron-sensing hormone hepcidin maintains iron balance by limiting iron availability through the inhibition of iron export from cellular stores when circulating iron levels exceed physiological thresholds [11,33–35]. At the cellular level, iron metabolism is controlled by iron regulatory proteins (IRPs), which bind to conserved iron-responsive elements (IREs) within the untranslated regions of mRNAs encoding the key iron-handling proteins, TfR1, DMT1, and FTH1 [33,36]. When iron levels are low, IRPs bind to IREs in the untranslated regions of mRNA, preventing the translation of ferritin and promoting the synthesis of TfRs. The IRE-IRPs system is not the sole mechanism regulating FTH1 expression. Indeed, FTH1 can also be degraded through a specific autophagic pathway known as ferritinophagy, first described by Mancias et al. [37]. Ferritinophagy involves the binding of Nuclear Receptor Coactivator 4 (NCOA4) to the FTH1 subunit of FTH, the delivery of the complex to the autophagosomes and its subsequent fusion with lysosomes to degrade FTH1 and release iron. Alternatively, they can be stored as hemosiderin or extracellularly released through endosomal microautophagy [38].

The tight regulation of iron availability, therefore, emerges as a critical determinant of cellular susceptibility to oxidative injury and ferroptotic cell death.

## 3. Ferroptosis

### 3.1. Molecular triggers and execution pathways

Ferroptosis is an iron-dependent form of RCD, not executed by caspases, driven by the lethal accumulation of phospholipid hydroperoxide (PL-OOH) within cellular membranes but rather stems from the catastrophic failure of cellular antioxidant defense systems to counterbalance metabolic oxidative stress [4,5,39]. The execution of ferroptosis relies on three critical and interdependent hallmarks: the availability of redox-active iron, the enrichment of membranes with polyunsaturated fatty acids (PUFAs), and the collapse of lipid peroxide defense mechanisms [40] (Figure 1).

**Figure 1.**
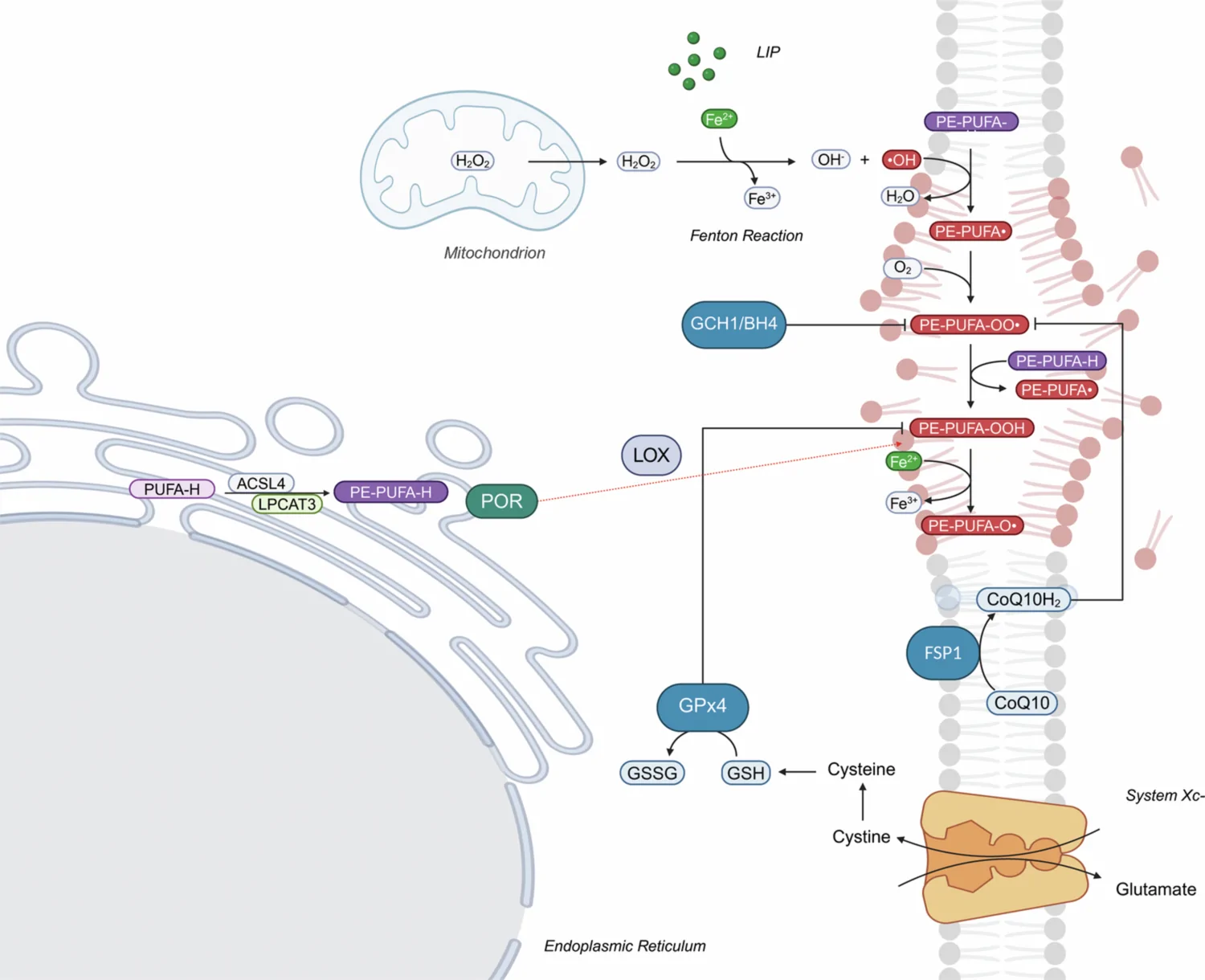
Ferroptosis initiation, radical chain amplification, and cytosolic defense mechanisms.

Central to this process is LIP, which provides the redox-active Fe^2+^ required to initiate oxidative cascades [41]. In the cardiac context, where iron homeostasis is tightly coupled to oxidative phosphorylation and mitochondrial bioenergetics, its dysregulation, as occurs during hemodynamic overload or post-infarction remodeling [42], creates a permissive environment for ferroptotic execution. In cardiomyocytes, mitochondria contribute significantly to cellular susceptibility to ferroptosis through two converging mechanisms: they are the predominant source of superoxide (O₂•^−^) via the ETC, and they harbor a discrete mtLIP that sustains localized Fenton chemistry. The ETC continuously generates O₂•^−^, which is rapidly dismutated into hydrogen peroxide (H₂O₂). Within the mitochondrial matrix, in the presence of mtLIP, H₂O₂ participates in Fenton chemistry, yielding highly reactive hydroxyl radicals (•OH) that initiate lipid peroxidation by abstracting hydrogen atoms from PUFA-rich phospholipids (Figure 2). Among these, cardiolipin, a mitochondria-specific phospholipid crucial for membrane stability and respiratory complex organization, represents a particularly vulnerable substrate for site-specific oxidative damage. Owing to its neutral charge and high membrane permeability, H₂O₂ further acts as a diffusible second messenger, escaping the mitochondrial matrix and entering the cytosol, where it encounters the LIP in close proximity to the plasma membrane and the ER (Figure 1). This spatial convergence triggers a secondary, widespread Fenton reaction, generating cytosolic •OH radicals that extend the peroxidative cascade to membrane-bound PUFAs throughout the cell.

**Figure 2.**
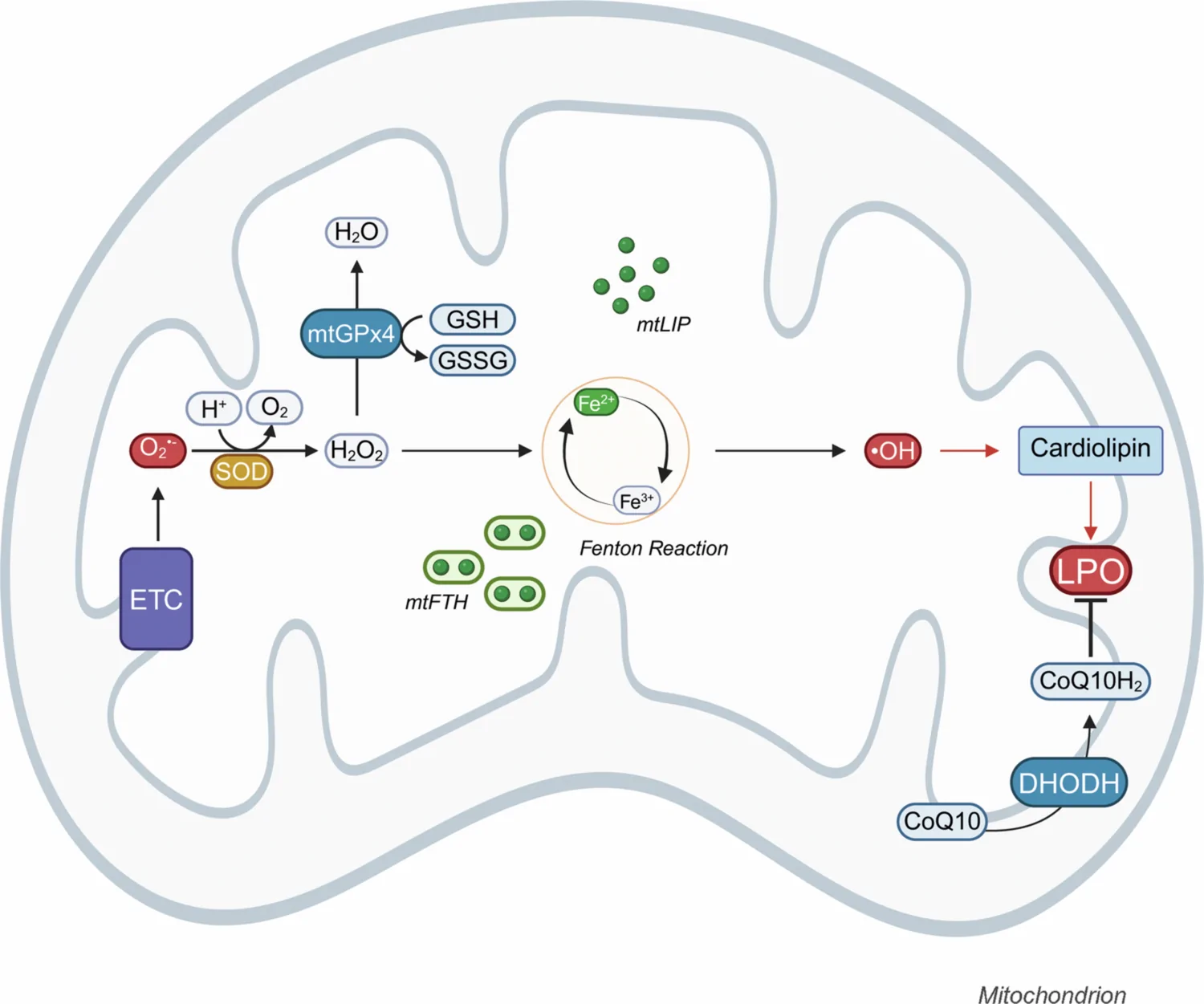
Mitochondrial pathways of lipid peroxidation and localized antioxidant systems.

The iron-driven oxidative cascade is further amplified by multiple self-reinforcing mechanisms. Fe^2+^ can directly decompose nascent PL-OOH into phospholipid alkoxyl radicals (PLO•), establishing a self-propagating chain reaction that exponentially accelerates membrane lipid peroxidation and generates secondary electrophilic products [43]. Concurrently, this process is not merely a stochastic chemical event driven by ROS overload, but is actively orchestrated by dedicated enzymatic machinery. Lipoxygenases (LOXs) catalyze the stereospecific oxygenation of PUFA-phosphatidylethanolamines (PEs), producing discrete hydroperoxy-lipid species that function as pro-death signals rather than random oxidative byproducts [44], while the cytochrome P450 oxidoreductase (POR) axis drives a parallel, iron-dependent peroxidative program at the ER membrane, further contributing to ferroptotic lipid peroxidation [45]. Notably, LOX-mediated peroxidation alone is sufficient to trigger the full ferroptotic cascade, and its pharmacological or genetic inhibition significantly attenuates cell death [44].

Beyond the availability of iron and enzymatic activity, the specific lipidomic composition of cellular membranes constitutes a fundamental determinant of ferroptotic susceptibility. The principal executioners are PEs esterified with arachidonic or adrenic acid, which represent the most oxidation-prone substrates within the lipid bilayer [46]. Cellular sensitivity to ferroptosis is therefore governed by a dynamic process of lipid remodeling orchestrated by ACSL4 (Acyl-CoA Synthetase Long-Chain Family Member 4) and LPCAT3 (Lysophosphatidylcholine Acyltransferase 3): ACSL4 activates arachidonic and adrenic acids to their coenzyme A (CoA) forms, which are subsequently incorporated into the membrane PE by LPCAT3, thereby establishing the pro-ferroptotic lipidomic signature required for cell death execution [47,48]. The indispensable role of ACSL4 in this program is underscored by the marked resistance to ferroptosis exhibited by ACSL4-deficient cells even under conditions of severe oxidative stress [49]. Critically, the preferential localization of ACSL4 at the ER and at MAMs positions these contact sites as a ‘ferroptotic synapse’, a spatially confined hub where lipid remodeling is directly coupled to mitochondrial ROS production, ensuring that oxidative stress is efficiently directed toward the vulnerable lipid pool to execute cell death [50–52]. Rather than a strictly sequential cascade, MAM tethering, ACSL4/LPCAT3-driven enrichment of PUFA-containing phospholipids, and mitochondrial ROS efflux operate as spatially coupled, mutually reinforcing processes concentrated at the ER–mitochondria interface. Within this framework, the physical architecture of the contact site itself may actively tune ferroptotic sensitivity, as tighter and more abundant contacts would be expected to shorten ROS diffusion distances and locally concentrate reactive flux at ACSL4-enriched membranes, a model that remains amenable to direct experimental verification. Collectively, the convergence of redox-active iron, enzymatic lipid peroxidation, and a pro-ferroptotic membrane composition culminates in the lethal accumulation of PL-OOH that drives ferroptotic execution in metabolically stressed cardiomyocytes, setting the stage for the equally sophisticated network of defense mechanisms that opposes this process.

### 3.2. Cellular-intrinsic protective mechanisms against ferroptotic cell death

Under physiological conditions, the peroxidative threat orchestrated by iron and polyunsaturated fatty acids (PUFAs) is counterbalanced by a robust and redundant network of antioxidant defense systems (Figure 1). The primary ‘gatekeeper’ of this network is the Glutathione Peroxidase 4 (GPx4) axis. GPx4, a selenoprotein, utilizes reduced glutathione (GSH), synthesized via the system Xc^−^ cystine/glutamate antiporter, to catalyze the reduction of toxic PL-OOH into chemically inert alcohols (PL-OH), thereby preserving membrane integrity. The critical nature of this axis is highlighted by the fact that direct pharmacological inhibition of GPx4 (e.g. by RSL3) or indirect GSH depletion through system Xc^−^ inhibition (e.g. by erastin) triggers uncontrolled lipid peroxidation and ferroptosis [53]. This vulnerability is particularly pronounced in cardiomyocytes because of their high metabolic demand and the specific enrichment of PUFAs within their mitochondrial and ER membranes [54]. However, recent discoveries have identified GPx4-independent surveillance systems that provide vital metabolic redundancy, ensuring cell survival even when the primary axis is compromised. The Ferroptosis Suppressor Protein 1 (FSP1)– ubiquinone (CoQ10) axis functions as a potent parallel defense mechanism. FSP1 is recruited predominantly to the plasma membrane via *N*-terminal myristoylation, where it catalyzes the NADPH-dependent reduction of CoQ10 to ubiquinol (CoQ10H₂). Ubiquinol acts as a lipophilic radical-trapping antioxidant (RTA) that directly intercepts lipid peroxyl radicals at membrane surfaces, halting the propagation of oxidative chain reactions independently of the GSH-GPx4 system [55]. In addition, the GTP cyclohydrolase 1-tetrahydrobiopterin (GCH1–BH4) axis has emerged as a specialized defense layer. GCH1 generates BH4, a potent RTA that specifically protects phospholipids with multiple polyunsaturated fatty acyl tails. The GCH1–BH4 axis offers a unique, tail-specific protection that preserves membrane fluidity and integrity even under conditions of severe oxidative stress [56,57].

## 4. Ferroptosis in cardiovascular diseases

CVDs encompass a broad spectrum of disorders involving the heart and blood vessels, characterized by progressive cardiomyocyte injury, which constitutes the main cellular population of the heart by volume. CVDs are the leading cause of death worldwide, and according to recent estimates of the World Health Organization, 19.8 million people die from CVDs each year [58]. Historically, apoptosis has been considered the principal form of regulated cardiomyocyte death, yet recent studies have increasingly recognized ferroptosis as a critical contributor to CVDs development [59,60].

The mechanistic framework outlined in the preceding sections, encompassing mitochondrial iron dysregulation, ER stress-driven lipid peroxidation, and the collapse of multi-organelle antioxidant defenses, finds its most clinically consequential expression in the cardiovascular system. The heart's exceptional dependence on mitochondrial oxidative phosphorylation, combined with the high PUFA content of cardiomyocyte membranes and the chronic oxidative burden imposed by hemodynamic stress, renders it uniquely susceptible to ferroptotic execution.

### 4.1. The regulatory role of ER

#### 4.1.1. ER signaling in ferroptosis

The ER is a network of branching, interconnected membranes composed of a single lipid bilayer that forms tubules and sacs, enclosing a narrow space known as the ER lumen. The primary function of the ER is to contribute to the synthesis and proper folding of a significant portion of the cell's proteins. In addition, being the main component responsible for Ca^2+^ storage, the ER plays a crucial role in Ca^2+^ homeostasis, and is actively involved in the biosynthesis, turnover and metabolism of lipids and steroids [61–63]. When the folding capacity of the ER is overwhelmed, unfolded or misfolded proteins accumulate within the lumen, triggering a state of ER stress and activating the unfolded protein response (UPR), an adaptive mechanism designed to alleviate the stress [64]. The UPR works primarily by altering gene expression and inhibiting protein synthesis to restore ER homeostasis. The three main ER stress sensors are localized in the ER membrane and include the inositol-requiring protein 1α (IRE1α), protein kinase RNA-like ER kinase (PERK), and activating transcription factor 6 (ATF6) [65]. Under normal conditions, these sensors remain inactive because of their association with the chaperone binding immunoglobulin protein (BiP). However, when unfolded proteins accumulate, they can competitively bind to BiP, releasing the sensors and thereby triggering the activation of UPR. In the context of CVDs, prolonged or unresolved ER stress has emerged as a critical sensitizer of ferroptotic signaling, acting through the remodeling of the cellular redox balance, lipid metabolism, and antioxidant capacity in ways that lower the threshold for ferroptotic execution [66].

Beyond its role in protein and lipid homeostasis, the ER has emerged as a critical platform for ferroptotic cell death regulation. The ER membrane is particularly enriched in PUFA-containing phospholipids and hosts the enzymatic machinery required for their peroxidation, positioning it as a key, though not exclusive, site of lipid peroxidation during ferroptosis, from which oxidative damage subsequently propagates to the plasma membrane [67]. A central driver of this process is POR. The POR–CYB5R1 axis has been identified as a potent enzymatic initiator of lipid peroxidation: POR and CYB5R1 transfer electrons from NAD(*P*)H to molecular oxygen, generating H₂O₂, which subsequently reacts with Fe^2+^ via Fenton chemistry to produce •OH that abstracts bis-allylic hydrogen atoms from PUFA-phospholipid chains, initiating the formation of PL-OOH [45]. Consistent with this dual role in stress signaling and lipid peroxidation, growing evidence positions ER stress and ferroptosis as functionally interconnected processes [68–71]. The ER is not merely a site of ferroptotic vulnerability but also contributes actively to cellular resistance against lipid peroxidation. A critical node of this defense is the availability of reduced GSH within the ER-proximal cytosolic compartment, which depends on the adequate supply of cysteine imported as cystine via the system Xc^-^ antiporter. The serine biosynthetic pathway has emerged as an important auxiliary source of GSH precursors: the rate-limiting enzymes phosphoglycerate dehydrogenase (PHGDH) and phosphoserine phosphatase (PSPH) support one-carbon metabolism and trans-sulfuration, thereby sustaining intracellular GSH levels and the activity of key antioxidant enzymes [72–74]. This metabolic axis is of particular relevance at the ER, where GSH availability directly determines the capacity of the GPx4 pool associated with ER membranes to detoxify nascent PL-OOH generated. Additionally, the ER membrane lipid composition itself can modulate ferroptotic susceptibility: remodeling of ER phospholipids toward reduced PUFA incorporation, governed upstream by ACSL4 and LPCAT3 activity, represents a potential cell-intrinsic mechanism to limit the availability of peroxidation-prone substrates at this site. Collectively, these ER-proximal defense mechanisms operate in parallel with the cytosolic GPx4 and FSP1 axes, underscoring the compartment-specific architecture of the cellular antioxidant network.

#### 4.1.2. ER stress as a bidirectional regulator of ferroptosis in CVDs

Unresolved ER stress has been shown to exert detrimental effects on cellular function and can act as an upstream contributor to ferroptosis in various CVDs. Recent pharmacological evidence further supports a mechanistic link. In a mouse model of aortic dissection, attenuation of ER stress, reflected by reduced activation of key UPR sensors such as PERK and IRE1α, mediates the protective effects of silibinin, the active compound of Silybum marianum [75]. This effect is accompanied by upregulation of the serine biosynthetic pathway, previously characterized as an ER-proximal defense mechanism supporting GSH availability, with restoration of the intracellular GSH pool and antioxidant enzyme activity [72–74]. This metabolic rewiring highlights a functional link between ER stress signaling and the regulation of ferroptotic susceptibility. Consistently, the bioactive compound dioscin, present in various medicinal plants, has been shown to attenuate ferroptotic damage in both cardiomyocytes and experimental models of myocardial infarction by simultaneously suppressing ER stress through inhibition of the PERK–eIF2α–ATF4 axis [76]. Although these findings demonstrate a concomitant reduction of ER stress and ferroptosis – consistent with, though not conclusive of, a functional contribution of ER stress to ferroptotic cell death – the precise molecular mechanisms underlying this protective effect remain unclear. Evidence for ER stress acting upstream of ferroptosis is especially compelling in obesity-associated cardiac remodeling [77], where ER stress acts as a predisposing factor for ferroptosis, which in turn propagates mitochondrial dysfunction and myocardial damage. Accordingly, treatment with the chemical chaperone TUDCA alleviates ER stress, reduces lipid peroxidation, and preserves mitochondrial structure and function in obese mice. Notably, these protective effects are abolished upon exposure to palmitic acid, a potent inducer of ER stress, underscoring the causal role of ER stress in driving ferroptosis-dependent cardiomyocyte dysfunction.

However, ER dysfunction is frequently regarded as a downstream consequence of ferroptotic stress, culminating in the activation of ER stress responses and UPR. Rather than being a passive bystander, this response actively contributes to the amplification of ferroptotic signaling by exacerbating redox imbalance and perturbing lipid homeostasis, ultimately reinforcing the progression toward cell death. This is exemplified by doxorubicin (DOX)-induced cardiac injury (DIC) [78], where DOX-induced ferroptosis occurring via GPx4 impairment/depletion in cardiomyocytes was demonstrated to be paralleled by activation of ER stress markers. In this context, loss of the endosomal trafficking regulator sorting nexin 3 (SNX3), previously implicated in the regulation of inflammation and oxidative stress pathways [79], conferred protective effects against doxorubicin-induced cardiotoxicity. These protective effects were reversed by pharmacological induction of ferroptosis with erastin, indicating that activation of the ferroptotic pathway in SNX3-deficient cardiomyocytes restored susceptibility to injury and led to the upregulation of ER stress markers following engagement of the ferroptotic program. Taken together, these findings suggest that SNX3 deficiency confers cardioprotection, at least in part, by dampening ER stress responses and attenuating ferroptotic signaling, mitochondrial dysfunction and cellular injury. Although a direct causal relationship cannot be definitively established, the data support a model in which ER stress occurs downstream of ferroptosis and acts to amplify the resulting cellular damage. The complex interplay between ferroptotic signaling and ER homeostasis was also highlighted in the context of atherosclerosis [80], where abnormal lipid metabolism was demonstrated to induce ferroptosis potentially through the modulation of ER function.

Collectively, the current evidence supports a model in which ER stress and ferroptosis engage in a self-reinforcing loop: unresolved ER stress lowers the ferroptotic threshold by depleting antioxidant capacity and remodeling the lipid landscape, while ferroptosis-driven membrane peroxidation disrupts ER proteostasis and amplifies stress signaling. The relative dominance of each direction appears to be context- and stimulus-dependent, with important implications for the interpretation of pharmacological interventions targeting either pathway in CVDs. Importantly, ER ferroptotic signaling does not occur in isolation but is deeply embedded within a broader inter-organelle network, in which mitochondria serve as both primary ROS sources and critical modulators of ferroptotic susceptibility.

### 4.2. The regulatory role of mitochondria

#### 4.2.1. Mitochondrial signaling in ferroptosis

Mitochondria are double-membrane organelles consisting of a highly folded IMM and an enclosing OMM. Beyond their canonical role in ATP production, they act as central hubs coordinating Ca^2+^ signaling, redox homeostasis, and iron metabolism, thereby critically influencing cellular fate [81–84]. The central role of mitochondria in the ferroptosis pathway, through ETC-derived superoxide, mtLIP-driven Fenton chemistry, and site-specific cardiolipin peroxidation, positions these organelles as a critical determinant of ferroptotic vulnerability. To counteract this peroxidative threat, mitochondria harbor a network of specialized antioxidant defense systems, including the mitochondrial GSH/GPx4 axis (mtGSH/mtGPx4), mtFTH1, superoxide dismutase 2 (SOD2), and the IMM-anchored enzyme dihydroorotate dehydrogenase (DHODH), which catalyzes the NADPH-independent reduction of CoQ10 to ubiquinol (CoQ10H₂) providing a GSH-independent radical-trapping defense that operates synergistically with mtGPx4 [85,86].

Evidence supporting mitochondrial involvement in ferroptosis is provided by the characteristic morphological changes these organelles undergo during the process, which serve as a defining hallmark of this form of cell death [4]. Ferroptotic cells display a distinctive mitochondrial phenotype (shrinkage, increased membrane density, and OMM rupture), while the nucleus remains relatively preserved, clearly distinguishing ferroptosis from apoptosis [4]. This distinct morphology underscores the indispensable role of mitochondrial structural integrity in ferroptotic progression.

Mitochondria have been shown to differentially contribute to the ferroptotic pathway, with mitochondrial metabolism being essential for cysteine deprivation, or erastin-induced ferroptosis, but largely dispensable for ferroptosis triggered by RSL3 [87,88]. Erastin acts as an inhibitor of the system Xc^-^ antiporter, thereby inducing ferroptosis as a consequence of cysteine depletion. As widely documented, cysteine plays a pivotal role not only in GSH synthesis but also in the biosynthesis of CoA and ISC [89–91]. Mitochondrial CoA and ISC availability critically sustain mitochondrial metabolism by enabling key enzymatic reactions within the tricarboxylic acid (TCA) cycle and the ETC [88]. Accordingly, cysteine deprivation leads to mitochondrial dysfunction and altered redox balance [88,92]. Consistently, cells lacking functional mitochondria display resistance to erastin, highlighting the dependence of erastin-induced ferroptosis on intact mitochondrial metabolism. In contrast, RSL3-induced ferroptosis bypasses the requirement for mitochondrial metabolism by directly inhibiting GPx4. Under these conditions, mitochondria are not essential for death initiation but instead act as modulators that amplify oxidative damage and influence the extent of ferroptotic injury. Indeed, restoring mitochondrial integrity and function has been shown to counteract RSL3-induced ferroptosis [93].

These findings position mitochondria as stimulus-dependent regulators of ferroptotic execution rather than obligate effectors, a distinction with direct implications for the interpretation of pathway-specific interventions in CVDs.

Collectively, the mitochondrial defense systems, operating through mechanistically distinct but functionally convergent pathways, constitute the terminal antioxidant layer that, together with the cytosolic GPx4, FSP1, and GCH1–BH4 axes described above, defines the multi-organelle resistance threshold against ferroptotic execution.

#### 4.2.2. Mitochondrial regulation of ferroptosis in CVDs

The mitochondrial ferroptotic axis can be conceptualized as a three-tiered system: iron influx and buffering (ZIP13/mtFTH1), gating and redox integration (VDAC1), and antioxidant execution (mtGSH/mtGPx4/DHODH). Perturbation at any tier, as occurs across a spectrum of CVDs, is sufficient to shift the mitochondrial redox setpoint toward ferroptotic vulnerability, positioning mitochondria not merely as passive targets but also as active determinants of cell fate in the ischemic, diabetic, and cardiotoxicity.

##### 4.2.2.1. Mitochondrial iron metabolism as a central determinant of ferroptotic vulnerability.

Mitochondria are central regulators of cellular iron metabolism, coupling iron homeostasis to redox regulation and, consequently, to ferroptotic vulnerability. The disruption of this balance has recently emerged as a key driver of ferroptotic cell death in the context of I/R injury. Notably, I/R has been shown to induce downregulation of ZIP13, which, together with impairment of the system Xc^−^/GSH/GPx4 antioxidant axis, is associated with defective mitochondrial ISC biosynthesis, accumulation of mitochondrial Fe^2+^, and the onset of ferroptosis in cardiomyocytes [94]. Within this framework, mtFTH1 acts as a critical regulator of mitochondrial iron homeostasis by limiting the expansion of the mtLIP. Consistently, the natural flavonoid Alpinetin (ALPT) has been shown to directly interact with and stabilize mtFTH1, thereby preserving the mitochondrial iron balance and attenuating both ferroptosis and apoptosis in cellular models of I/R injury [95].

These findings identify mitochondrial iron dyshomeostasis as a central determinant of ferroptotic susceptibility in I/R injury, positioning the mitochondrial iron buffering systems as a critical modulators of cardiomyocyte fate in CVDs.

##### 4.2.2.2. VDAC1 as an integrative gateway between mitochondrial iron handling and antioxidant defense.

Voltage-dependent anion channel 1 (VDAC1), the principal channel of the OMM, mediates bidirectional metabolite and ion trafficking between the cytosol and the mitochondrial compartment. Emerging evidence suggests that VDAC1, beyond its transport role, may function as a regulatory node that integrates mitochondrial iron homeostasis and antioxidant capacity, thereby occupying a pivotal position between the pro-ferroptotic iron axis and the defensive GPx4 axis described below.

In I/R models, alterations in VDAC1 signaling are functionally coupled to changes in mtFTH1-dependent iron buffering capacity [96]: VDAC1 upregulation during anoxia/reoxygenation (A/R) injury promotes mitochondrial dysfunction, an effect that is reversed by increased mtFTH1 levels, which attenuate VDAC1 activity and restore mitochondrial homeostasis. This functional coupling between VDAC1 and mtFTH1 identifies a regulatory circuit in which the iron buffering capacity directly modulates the permeability state of the OMM. Concurrently, VDAC1 influences the mitochondrial redox environment in a manner that compromises GPx4 functionality: during A/R injury, VDAC1-dependent perturbation of the redox landscape impairs mtGPx4 activity, whereas resveratrol-induced downregulation of VDAC1 is associated with preservation of GPx4 function, reduced lipid peroxidation, and attenuation of ferroptotic cell death [97,98]. Collectively, these findings establish VDAC1 as a putative bidirectional checkpoint that amplifies ferroptotic vulnerability when upregulated by expanding mtLIP and impairing GPx4 function, and confers protection when downregulated or functionally restrained, thereby shielding the mitochondrial compartment from oxidative collapse. However, the extent to which VDAC1 directly coordinates these processes, as opposed to reflecting broader mitochondrial stress responses, remains to be fully elucidated.

##### 4.2.2.3. The mitochondrial GSH/GPx4 and DHODH axes as regulators of ferroptosis in cardiovascular disease.

Downstream of the iron-regulatory circuit governed by mtFTH1 and VDAC1, the mitochondrial antioxidant network constitutes the terminal defense layer against ferroptotic lipid peroxidation. The mtGSH/mtGPx4 axis is increasingly recognized as a key effector of mitochondrial antioxidant defense in CVDs: activation of mtGPx4 reduces lipid hydroperoxides and limits the accumulation of DOX–Fe^2+^ complexes within mitochondria, thereby mitigating ferroptotic damage [99]. The functional integrity of this axis critically depends on the availability of mitochondrial GSH, which is maintained by the SLC25A11 transporter. Notably, DOX promotes the interaction between FUN14 domain-containing 2 (FUNDC2), a conserved OMM protein, and SLC25A11, leading to impaired GSH import, elevated mitochondrial ROS, and accelerated lipid peroxidation [100]. In I/R models, pretreatment with Morinda officinalis oligosaccharides (MOO) restores mtGPx4 levels and enhances SOD2 activity through the inhibition of NADPH oxidase NOX4, collectively attenuating oxidative injury in cardiac tissue and isolated cardiomyocytes [101]. Consistently, in diabetic cardiomyopathy, the mitochondria-targeted antioxidant SS-31 activates the mtGSH/mtGPx4 axis, reduces mitochondrial Fe^2+^ accumulation, and suppresses mitochondria-dependent ferroptosis [102].

Complementing this glutathione-dependent axis, DHODH provides a parallel, GSH-independent line of defense [103]. The convergence of mtGPx4 and DHODH on the mitochondrial lipid peroxidation threshold, through mechanistically independent but functionally synergistic pathways, creates a layered defense architecture that is of particular relevance in the myocardium, given the intense bioenergetic demands and the high ubiquinone cycling flux of cardiac tissue.

Collectively, these functional properties described above, combined with their intimate structural association with the ER at MAMs, place mitochondria at the nexus of the inter-organelle ferroptotic network discussed in the following section.

### 4.3. Do MAMs define ferroptotic susceptibility in CVDs? Emerging mechanisms and functional implications

The mitochondrial and ER membranes are tightly juxtaposed, forming reversible dynamic contact regions approximately 10–50 nm wide that facilitate inter-organelle communication. MAMs orchestrate Ca^2+^ homeostasis, mitochondrial dynamics, and metabolic signaling pathways, including lipid trafficking and biogenesis [104–106] (Figure 3). These processes are mediated by multiprotein complexes that, rather than functioning merely as anchoring platforms, couple structural tethering with specific ferroptosis-relevant biochemical activity, including ion transfer, redox sensing, or lipid translocation. This finding underscores that their contribution to ferroptotic susceptibility derives from their function exerted at the contact site itself, rather than merely from ER or mitochondrial residency. Among the best-characterized MAMs functional components is VDAC, which, together with the inositol 1,4,5-trisphosphate receptor (IP3R) on the ER, the chaperone glucose-regulated protein 75 (Grp75) and MCU on the IMM, form a signaling bridge that mediates Ca^2+^ transfer from the ER to the mitochondria. Although Ca^2+^ transfer through the VDAC–MCU axis is well established, emerging evidence suggests that VDAC1 may play a broader regulatory role extending to the modulation of mitochondrial iron trafficking and homeostasis [107–109]. These observations raise the possibility that VDAC1 serves as a multifunctional gateway coordinating both Ca^2+^ and iron homeostasis across the OMM, thereby linking ion homeostasis to ferroptotic vulnerability [96,98].

**Figure 3.**
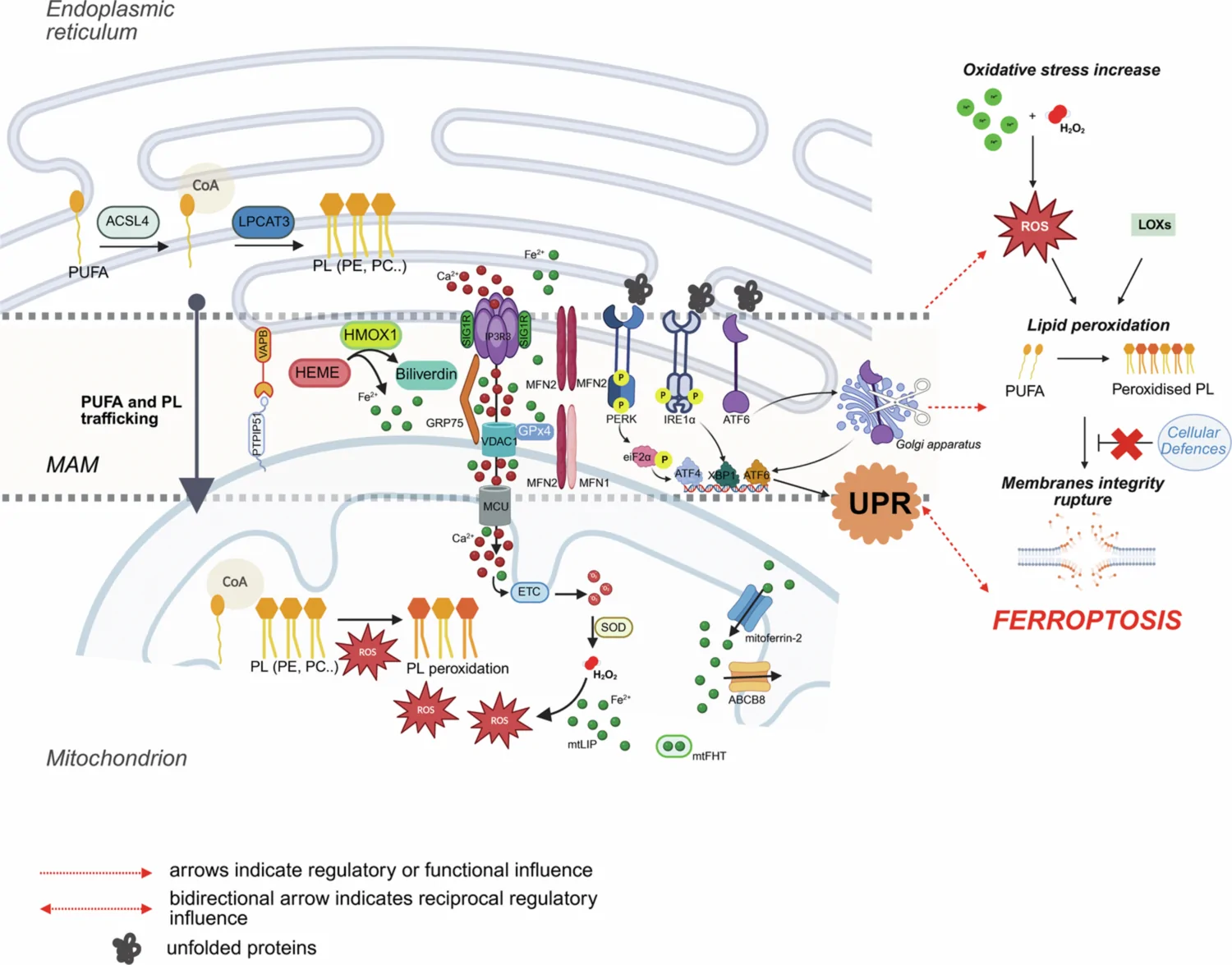
Central role of mitochondria-associated membranes (MAMs) in ferroptosis.

Beyond ion exchange, MAMs also function as critical hubs for stress signaling, by undergoing dynamic structural and proteomic remodeling, characterized by the transient accumulation and reorganization of specific proteins, as evidenced by the enrichment of ER stress sensors such as PERK and IRE1α, which contribute to the formation of specialized microdomains characterized by increased oxidative stress and metabolic vulnerability [110,111]. In this context, the voltage-dependent anion channel isoform 3 (VDAC3) has emerged as an additional stress-responsive component, although whether these functions are specifically exerted at MAMs remains to be established [112]. Notably, its cysteine residues act as redox-sensitive switches, enabling VDAC3 to sense oxidative perturbations and mitigate mitochondrial ROS accumulation, thereby preserving mitochondrial functionality [112]. Adaptive MAMs remodeling under oxidative burden is clearly illustrated by the RMDN3/PTPIP51 (regulator of microtubule dynamics 3) -VAPB (VAMP-associated protein B and C) tethering complex, which responds to mitochondrial ROS by increasing ER-mitochondria contacts. The tetratricopeptide repeat (TPR) domain of RMDN3 then transfers lipid radicals from the mitochondria to the ER, preventing their accumulation and subsequent cell death [7]. This adaptive remodeling enables MAMs to detect perturbations in cellular homeostasis and to transduce these signals into coordinated ER–mitochondrial responses.

Accumulating evidence has firmly established the role of HMOX1 as a critical and functionally paradoxical regulator of ferroptotic susceptibility in cardiomyopathy [113–115]. While PERK, IRE1α, and VDAC3 primarily sense oxidative and ER stress, HMOX1 represents a downstream stress-responsive effector that may translate these signals into ferroptotic susceptibility by reshaping local iron and redox homeostasis. By catalyzing heme degradation, HMOX1 generates the antioxidant metabolites biliverdin and its lipid-soluble derivative bilirubin, alongside Fe^2+^. This liberated iron amplifies oxidative stress through Fenton chemistry, promoting lipid peroxidation and positioning HMOX1 at the intersection between cytoprotective and pro-ferroptotic functions, with the net outcome depending critically on the balance between iron liberation and antioxidant metabolite production in a given pathological context [116].

Although HMOX1 is predominantly localized at the ER, it can redistribute to other membrane compartments under conditions of oxidative or metabolic stress, suggesting stress-dependent shuttling between the ER and mitochondrial compartments with MAMs representing a likely route for HMOX1 trafficking [117]. Whether the ferroptosis-related effects of HMOX1 specifically rely on its recruitment to MAMs remains to be established; however, these contact sites could serve as a spatially restricted microenvironment in which the principal determinants of ferroptosis converge: HMOX1-derived Fe^2+^, mitochondria-derived ROS, and ER-derived PUFA-containing phospholipids susceptible to peroxidation. At the same time, the local production of biliverdin and bilirubin may provide an intrinsic antioxidant counterbalance, suggesting that HMOX1 positioned at the ER–mitochondria interface could function as a context-dependent rheostat that determines whether oxidative stress is contained or amplified into ferroptotic execution. Consistent with this model, HMOX1 has been implicated in ferroptosis across multiple cardiovascular settings, including atherosclerosis, diabetic cardiomyopathy, and ischemia/reperfusion injury. In atherosclerosis, HMOX1 promotes ferroptotic death of the lipid-associated pro-atherogenic TREM2-low foamy macrophages within plaques through its interaction with lactate dehydrogenase B (LDHB), facilitating Lon Peptidase 1 (LONP1)-dependent degradation of mitochondrial transcription factor A (TFAM), thereby inducing mitochondrial dysfunction and ferroptotic cell death [118]. In DIC, transcriptomic analysis revealed that HMOX1 upregulation drives lipid peroxidation of mitochondrial membranes, a process attenuated by the mitochondria-targeted antioxidant MitoTEMPO [119]. In I/R injury, HMOX1 activation promotes iron accumulation within the ER, further amplifying ferroptotic susceptibility through ER-localized oxidative damage [120]. While the functional contribution of HMOX1 redistribution to MAMs in ferroptotic signaling in CVDs remains to be established, the discussed findings support the notion that HMOX1 may act as a context-dependent modulator of ferroptotic susceptibility, potentially linking iron metabolism, mitochondrial dysfunction, and ER stress at the ER–mitochondria interface.

Besides coordinating stress signaling, MAMs are constitutively enriched in lipid-metabolizing enzymes that directly shape the ferroptotic lipid landscape. Among these, VDAC itself has recently been identified as a phospholipid scramblase: dimers of VDAC1 and VDAC2 catalyze rapid transbilayer translocation of phospholipids across the OMM at a specific dimer interface, providing the main pathway by which ER-derived phospholipids, including PUFA-containing species, are imported into mitochondria [121,122]. This scramblase function is mechanistically distinct from VDAC's channel activity and positions VDAC as a direct conduit linking ER-derived lipid metabolism to mitochondrial membrane composition, with implications for the availability of ferroptosis-prone substrates at the OMM [121]. Importantly, this lipid-translocating activity integrates with VDAC’s established role as a structural component of MAMs, where it contributes to ER–mitochondria tethering and inter-organelle lipid exchange, as well as with its previously discussed function as a ferro-redox gateway coordinating metabolite fluxes, iron handling, and redox signaling across the OMM. Together, these convergent functions place VDAC at the interface between membrane remodeling, iron-dependent oxidative metabolism, and ferroptotic susceptibility. Complementing this lipid import function, MAMs are further enriched in phosphatidylserine synthases 1 and 2 (PSS1 and PSS2), consistent with the localization of key steps of phospholipid biosynthesis and remodeling at the cytosolic face of the ER membrane [123]. Additionally, MAMs formation has been shown to regulate ACSL4-dependent phospholipid remodeling, thereby suggesting a possible role of ER–mitochondria contact sites as functional hubs of ferroptosis-relevant lipid metabolism [50,124].

Emerging evidence suggests that dynamic remodeling of the ER–mitochondria interface represents an additional regulatory layer through which MAMs influence ferroptotic susceptibility by integrating changes in organelle architecture, lipid metabolism, and redox homeostasis.

Highlighting the putative position of MAMs as integrative platforms linking mitochondrial and ER dynamics to lipid peroxidation in ferroptosis, recent evidence indicates that the transcriptional regulator SAP30-binding protein (SAP30BP) exacerbates high glucose–induced ferroptosis in cardiomyocytes through modulation of the mitofusin 2 (MFN2)–ACSL4 axis [125]. Given the established role of MFN2 as a key structural component of MAMs and the localization of ACSL4 to the ER and MAMs compartments [51,126], these findings suggest that MAM remodeling can couple mitochondrial dynamics with ACSL4-dependent phospholipid remodeling during ferroptosis. The essential contribution of ACSL4 to the regulation of ferroptosis in CVDs was further substantiated in pressure overload-induced cardiomyopathy in mice, where its upregulation exacerbates cardiac dysfunction and promotes the development of heart failure [127].

Dynamic structural remodeling of the ER–mitochondria interface also plays a fundamental role in ischemic injury [128]. This is exemplified in acute myocardial infarction, where ischemia-induced activation of the ER stress sensor PERK disrupts its interaction with MFN2, leading to MAMs remodeling, altered mitochondrial redox balance, and the promotion of ferroptotic cell death [129].

Furthermore, beyond their roles in signal integration and stress sensing, MAMs have recently been identified as early hotspots of phospholipid peroxidation, where lipid oxidative damage is initiated and propagated, thereby directly driving ferroptotic cell death [8]. Nevertheless, the impact of MAM remodeling on ferroptosis remains incompletely understood and appears to be highly context-dependent. On the one hand, enhanced ER–mitochondria contacts have been shown to facilitate RSL3-induced ferroptotic execution [8]. On the other hand, pathological disruption of MAM integrity during ischemic stress has also been reported to promote ferroptosis by impairing mitochondrial homeostasis and increasing mitochondrial ROS production [129]. While the data presented here indicate that MAMs are not merely passive structural platforms, it remains to be fully elucidated whether their physical parameters, such as intermembrane distance, contact abundance, and subcellular localization, actively tune ferroptotic signaling. Thus, MAMs should not be considered intrinsically pro- or anti-ferroptotic structures; rather, their functional state, molecular composition, and remodeling dynamics determine whether they facilitate adaptive stress responses or promote ferroptotic execution.

Collectively, these observations support the concept that MAMs may function as critical decision-making hubs that integrate and relay stress signals between the ER and mitochondria, consistent with a model in which MAMs integrate metabolic and oxidative cues to set the threshold for ferroptotic cellular susceptibility.

However, despite growing evidence supporting their functional importance, several fundamental questions regarding MAMs remain unresolved in the cardiovascular context. The molecular composition of these contact sites under ferroptotic conditions, including which tethering complexes are remodeled, which lipid-metabolizing enzymes are recruited, and which redox-sensitive proteins are activated, has yet to be systematically characterized. Equally critical is the need to disentangle causal relationships from correlative observations: whether MAMs actively drive ferroptotic execution or instead reflect a secondary reorganization in response to upstream organelle stress remains unclear. Finally, determining whether these structures can be selectively targeted for therapeutic intervention in CVDs, without broadly disrupting the homeostatic functions they serve, represents a significant translational challenge that warrants dedicated investigation [130–132].

## 5. Concluding remarks and future perspectives

Preclinical evidence supporting the efficacy of anti-ferroptotic therapies has steadily increased across a range of pathologies, including CVDs [60]. Among the most extensively characterized compounds are the lipophilic radical-trapping antioxidants ferrostatin-1 and liproxstatin-1, as well as iron chelators such as deferoxamine (DFO) and deferiprone [97,133–136] (Table 1). Despite their robust activity in experimental models, their clinical translation remains limited by intrinsic pharmacological constraints, including short half-life, low bioavailability, chemical instability, and off-target effects [137–139]. Moreover, prolonged antioxidant treatment may interfere with the physiological clearance of damaged cells and disrupt tissue homeostasis.

**Table 1.** Preclinical ferroptosis inhibitors with cardioprotective potential.

Compound class	Molecule	CVDs conditions	Refs
**Radical-trapping antioxidants** **(RTAs)**	Ferrostatin-1 (Fer-1)	DIC, Pressure Overload–Induced Cardiomyopathy, Myocardial I/R injury	[134,140,141]
	Liproxstatin-1 (Lip-1)	I/R injury, Myocardial Infarction (MI)	[97,142]
	UAMC-3203	Heart Failure (HF), Aortic Dissection (AD)	[143,144]
**Iron chelators**	Deferoxamine (DFO)	I/R injury, Cardiac Arrhythmias	[145,146]
	Dexazoxane	DIC	[147]
	Deferiprone	Heart Failure, I/R injury	[148,149]
**Mitochondria-targeted agents**	MitoTEMPO	DIC	[119]
	MitoQ	Cardiac Hypertrophy	[150]
**Enzymatic inhibitors**	Baicalein	I/R injury, Myocardial Injury	[151,152]
**System xCT inducers**	*N*-Acetylcysteine (NAC)	I/R injury, Myocardial Injury	[153]
	Atorvastatin	Heart failure, I/R injury	[154,155]
	C3G	Myocardial injury	[156]
**Inhibitors of ferritinophagy**	Kaempferol (KP)	DIC	[157]
**Natural antioxidants targeting ferroptosis**	Morinda officinalis Oligosaccharides (MOO)	Myocardial I/R injury	[101]

To overcome these limitations, several next-generation compounds have been developed. For instance, the second-generation ferroptosis inhibitor UAMC-3203 exhibited improved stability, potency, and safety compared with ferrostatin-1 [158]. In parallel, emerging non-canonical radical-trapping antioxidants, such as *γ*-terpinene, have shown the ability to inhibit lipid peroxidation through distinct autoxidative mechanisms, although their in vivo efficacy remains to be established [159]. Targeting ferroptosis at the subcellular level represents an additional promising strategy: mitochondria-directed approaches, such as Mito-DFO/DFX and MitoTEMPO, enhance pathway specificity and may improve therapeutic safety [119,160]. Additionally, inhibition of the 15-lipoxygenase-2/phosphatidylethanolamine-binding protein 1 (15LOX-2/PEBP1) complex represents a promising strategy to prevent PUFA-PE peroxidation while preserving physiological lipoxygenase signaling. Although direct evidence in cardiovascular models remains limited, accumulating data support the involvement of this pathway in CVDs pathogenesis [161,162].

Within this framework, the interplay between mitochondria, ER, and MAMs is increasingly recognized as a central hub in the regulation of ferroptotic sensitivity. Dysregulation of these inter-organelle communication networks may therefore critically influence the susceptibility of cardiovascular cells to ferroptotic damage. Despite these advances, many of these compounds remain suboptimal for in vivo application, underscoring the need for more effective and pharmacologically robust anti-ferroptotic agents. Equally important is the identification of reliable and specific biomarkers capable of distinguishing ferroptosis from other forms of regulated cell death, which remains a major unmet need for the safe and effective clinical translation of these therapies. Looking forward, the identification of novel molecular targets within ferroptosis-regulating pathways, together with a deeper understanding of the role of MAMs in modulating cellular susceptibility to ferroptosis in cardiovascular contexts, may open new avenues for the development of innovative therapeutic strategies. Targeting the dynamic crosstalk between mitochondria and the ER at MAM interfaces holds particular promise, offering the potential to selectively modulate ferroptotic signaling while preserving essential cellular functions. Beyond the emerging pivotal role of MAMs as regulatory hubs of ferroptosis, it is important to recognize that mitochondria also establish dynamic contact sites with other subcellular organelles, including the nucleus and, most notably, lysosomes. Lysosomes play a central role in intracellular iron homeostasis by coordinating iron uptake, storage, mobilization, and recycling, while mitochondria–lysosome contact sites and ferritinophagy have emerged as complementary mechanisms controlling the intracellular labile iron pool and ferroptotic susceptibility across diverse pathological settings [28,163–165]. Future studies should therefore move beyond an exclusive focus on MAMs and investigate how multiple organelle contact sites cooperate to coordinate iron metabolism, lipid peroxidation, and stress signaling.

.
